# Prevalence and correlates of disordered eating among adults who received weight-related nutrition consultations

**DOI:** 10.1017/jns.2026.10100

**Published:** 2026-05-05

**Authors:** Luis Ortiz-Hernandez, Elizabeth Tapia-Hernandez

**Affiliations:** Health Care Department, Universidad Autónoma Metropolitana - Unidad Xochimilco, Mexico

**Keywords:** Binge eating, Dietetics, Disordered eating, Obesity, Weight management

## Abstract

Disordered eating (DE) is common among patients attending primary health care settings. However, the prevalence of DE among patients who receive care in nutrition practice settings has yet to be estimated. We aimed to determine the prevalence of DE and its correlates among outpatients in a nutrition service. A cross-sectional survey was conducted using a convenience sample of outpatients (*N* = 502) who received care from 2022 to 2024 at the Nutrition Care Offices, which is a university nutrition service in Mexico City. A screening questionnaire was created to identify DE. Items were derived from existing DE screening tools and patients’ experiences. Linear regression models were estimated, with the outcomes being the three indicators of DE (negative body image, binge eating-compensation, and exercise as a negative experience). The most common DE behaviours and cognitions were feeling uncomfortable or insecure about their body fat (74.7%), feeling ashamed of their weight (57.6%), feeling uncomfortable or insecure about their muscles (57.2%), feeling bad when their weight is measured (54.0%), and feeling they have lost control of what they eat (51.0%). Binge eating-compensation and negative body image scores were higher among women, younger individuals, those with higher body weight, and those with very light or light leisure-time physical activity (PA). The practice of leisure-time PA was positively associated with exercise as a negative experience but negatively related to negative body image. Our findings suggest that DE is a problem that arises recurrently in weight-related nutrition consultations. Higher risk groups deserved special attention.

## Introduction

One of the available fields of work for nutritionists is primary care. At this level, common reasons for people seeking nutritional care include intentions to lose weight or body fat, to gain weight or muscle mass, or to achieve both. The first reason for individuals to book a consultation related to weight loss can be attributed to lifestyle changes due to urbanisation, as well as the increase in the obesity rate and its comorbidities.^(1)^

At the same time, interest in reducing body fat and increasing muscle mass can be attributed to sociocultural shifts in what constitutes a ‘perfect body’. The term ‘diet culture’ has been proposed to describe the sociocultural norms that idealise thinness in women. However, given the increasing concern about muscularity and exercise, another more accurate term is the ‘perfect body culture’.^(2)^ This concept aims to recognise that there are sociocultural pressures on both women and men to focus on their physical appearance and seek modifications to conform to gender-specific stereotypes. In the Mexican context, the ‘perfect body’ for women is characterised by thinness with muscle tone, whereas for men, the stereotype is based on high levels of muscularity.

The perfect body culture has promoted an increase in body image concerns and disordered eating (DE) behaviours and thoughts. According to the Diet and Nutrition Academy,^(3)^ DE is defined as ‘beliefs, attitudes, thoughts, and behaviours related to food, eating, body composition, and weight, including classic eating disorders, as well as similar conditions that negatively impact health or quality of life.’ Examples of these thoughts and emotions include thin-ideal or muscular-ideal internalisation and body dissatisfaction, which can lead to restrictive behaviours (such as dieting, fasting, or avoiding high-density energy foods), overeating, and compensatory measures (such as excessive exercise, vomiting, or taking medications). DE is prevalent and can have negative consequences on health and well-being. Prospectively, DE is linked to psychological distress, poor self-rated health,^(4)^ and lower quality of life.^(5)^ In addition, DE is a risk factor for eating disorders (ED), such as anorexia nervosa, bulimia nervosa,^(6)^ and muscle dysmorphia.^(7)^ In Mexican adolescents, the most prevalent DE behaviours and cognitions are overeating (45.8%), concern about gaining weight (35.1%), loss of control over eating (35.1%), and excessive exercise for weight loss (14.1%).^(8)^

In patients who received medical care,^(9–12)^ the prevalence of DE ranged from 0.6% (self-induced vomiting) to 32.0% (overeating). However, these estimates are from the USA ^(9–11)^ and Australia^(12)^ and only include people with obesity^(11)^ or women.^(9,10)^ To date, no studies have been conducted on outpatient nutrition services in middle-income countries. It is expected that DE may be common among patients who are looking for weight change, since the rates of DE tend to be higher among people with overweight and obesity.^(13)^ Considering the above, the current study aimed to know the prevalence of DE among individuals who attended a university project focused on nutritional care for outpatients. Another objective of the study was to identify factors associated with DE.

## Methods

This article reports on the experience gained at the ‘Nutrition Care Offices’ (NCO, *Consultorios de Atención Nutricional*), a university nutrition service in Mexico City. In this service, students and interns from the Bachelor of Human Nutrition programme provide nutrition counselling to healthy individuals or those with medical conditions who receive care at the primary level.

An observational, cross-sectional study was conducted using data from adult patients who received care at the NCO from May 2022 to May 2024. The analysed data were extracted from questionnaires completed by all patients who received care at the NCO prior to their appointment. This pre-consultation questionnaire is a self-administered PDF form with two sections, and it is required to receive care. The first section collects information on the patient’s sociodemographic characteristics, health status, lifestyle, and reasons for consultation. The second section included 15 questions about DE. The patient is required to complete only the second section if a reason for their consultation is a change in her/his weight and/or body composition. Pre-consultation questionnaires that were not answered or had missing information about DE (*n* = 32) were discarded. Patients under 18 years of age (*n* = 6) and those who reported a reason for the consultation that was not related to weight (*n* = 82) were excluded. Data from patients with incomplete sociodemographic and lifestyle data were also discharged (*n* = 30). The final sample comprised 502 patients. In this way, only individuals with complete data were included in the statistical analyses.

### Ethics

This study was conducted in accordance with the guidelines set out in the Declaration of Helsinki, and all procedures involving patients were approved by the Divisional Council of Biological and Health Sciences of the UAM-X (agreements 1/19.10.1 and 7/23.5.2). Written informed consent was obtained from all patients.

### Questionnaire to screen for DE behaviours and cognitions

Since the beginning of the NCO (in 2010), a common reason for consultation has been the intention to lose weight. It was also observed that some patients who wanted to lose weight were concerned about body image and/or experienced binge eating. Sporadically, people with low weight and a reluctance to eat were identified. Most recently, concerns about muscularity and exercise were also evident in the consultations. Based on these observations, the NCO team considered it necessary to identify patients with DE. The rationale was that screening for DE^(14–16)^ could help avoid prescribing counterproductive interventions (e.g., restrictive diets)^(17,18)^ for patients with these conditions and, in necessary cases, refer them to appropriate care (e.g., mental health professionals).^(16)^ For this purpose, the screening tool should consider the variety of DE behaviours and cognitions that patients experience, i.e., body dissatisfaction, restrictive practices, overeating, compensatory measures, and/or excessive exercise. However, existing questionnaires^(19)^ consist of many items and focus on specific forms of DE. Therefore, it was necessary to develop a screening tool that imposed no undue burden on patients and encompassed their diverse experiences.

The questionnaire used to identify DE is presented in Table 1. Questions 1, 6, 8, 13, and 14 were formulated by the NCO project team, considering patients’ experiences. Other questions were selected and adapted from screening inventories to assess body-image concern and eating behaviours. Following the guidelines,^(14,16)^ the Spanish version of the SCOFF questionnaire^(20)^ was used (questions 3, 9, 10, 11, and 12 in Table 1). The phrase ‘or guilty’ was added to the original item of the SCOFF about self-induced vomiting (‘Do you make yourself vomit because you feel full?’, question 9). Item 61 of the Eating Disorder Inventory ^(19)^ was modified to include the phrase ‘or hiding from other people’ (question 7).

**Table 1.** Exploratory factor analysis of the questionnaire on disordered eating in patients from the Nutritional Care Offices, Mexico City (*N* = 502)

	Sources of items	Overall	Men	Women	Exploratory factor analysis			
Items		%	%	%	F1	F2	F3	F4
Eigen value					3.02	2.95	2.14	1.12
Variance, %					20.1	19.7	14.3	7.4
1. Do you feel nervous or bad when you weigh or measure yourself?	OR	54.8	34.5	62.8*	0.52			
2. Do you feel ashamed of your weight or body size?	MBAS	57.6	39.4	64.7*	0.69			
3. Do you think you are fat even though others say you are thin?	SCOFF	50.0	35.2	55.8*				
4. Do you feel uncomfortable or insecure about your muscles?	MBAS	57.2	47.9	60.8*	0.77			
5. Do you feel uncomfortable or insecure about your body fat?	MBAS	74.7	63.4	79.3*	0.67			
6. Does counting calories or macros or measuring or weighing food make you feel bad?	OR	19.5	10.6	23.1*				
7. Have you eaten in secrecy or hiding from other people?	EDI	24.7	7.8	31.4*		0.69		
8. Without being hungry, do you eat a lot in a short period of time (less than 2 hours)?	OR	35.7	28.9	38.3*		0.63		
9. Do you make yourself vomit because you feel full or guilty?	SCOFF	5.0	2.1	6.1		0.62		
10. Are you worried that you have lost control over the amount of food you eat?	SCOFF	51.0	35.9	56.9*		0.43		
11. Have you lost more than 7 kilos in the last 3 months?	SCOFF	5.4	4.9	5.6				0.87
12. Would you say that food dominates or controls your life?	SCOFF	19.7	14.8	21.7		0.59		
13. Do you feel like you are obsessed or worried about what you eat?	OR	39.0	28.2	43.3*		0.43	0.43	
14. Have you exercised to compensate for what you ate?	OR	43.0	45.1	42.2			0.77	
15. Do you feel anxious or depressed when you miss workout days	MDDI	44.0	47.2	42.8			0.80	

*Note:* %, proportion of affirmative answers.

* *p* < 0.050 for comparison between sexes.

OR, original items developed in the Nutritional Care Offices project; SCOFF, Sick, Control, One stone, Fat and Food questionnaire;^(20)^ EDI, Eating Disorder Inventory;^(19)^ MBAS, Male Body Attitudes Scale;^(21,22)^ MDDI, Muscle Dysmorphia Disorder Inventory.^(23)^

Items from other inventories were selected to assess concerns about muscularity and excessive exercise. Two questions (4 and 5 of Table 1) were formulated based on four questions of the Revised Male Body Attitudes Scale (MBAS-R).^(21)^ In the original items, the words ‘embarrassed’ and ‘ashamed’ are used. These were replaced by ‘uncomfortable or insecure’. Item 23 from the original version of the MBAS^(22)^ was used (question 2 in Table 1). Items 10 (‘I feel anxious when I miss one or more workout days’) and 12 (‘I feel depressed when I miss one or more workout days’) of the Muscle Dysmorphia Disorder Inventory (MDDI)^(23)^ were combined into a single question (number 15). The Spanish versions of the MBAS and MADDI have been previously used with Mexican gym users.^(24)^ To facilitate the completion of the questionnaire and homogenise the answers, the scales with Likert-type responses were converted to dichotomous response options (yes/no).

In an exploratory factor analysis with oblique rotation (*oblimin*), four factors emerged (Table 1). Only factor loadings equal to or greater than 0.40 were considered and reported. Item 11 was the only one included in the fourth factor; therefore, it was discharged. Questions 3 and 6 were not located in any of the factors. The first three factors explained 54.1% of the variance. The first factor was named ‘negative body image’ and consisted of four questions. The second factor, called ‘binge eating-compensation’, included six questions. The third factor was named ‘exercise as a negative experience’ and included three questions. Three variables were created with the sum of affirmative responses to the items included in each factor. These indicators of DE (negative body image, binge eating-compensation, and exercise as a negative experience) were considered outcomes. For the three variables, higher values indicate higher levels of the construct. The range of possible values for each variable was from 0 (indicating the absence of negative body image, binge eating-compensation, and exercise as a negative experience) to the number of items in each factor (4, 6, and 3, respectively). The Cronbach’s alpha values of all items and each factor were 0.80, 0.67, 0.71, and 0.56, respectively.

### Covariates

In the same pre-consultation questionnaire, the following patients’ data were requested: self-reported weight and height, sociodemographic characteristics (sex, age, and education), and lifestyle factors (tobacco and alcohol use and physical activity). The age groups created were 18 to 20, 21 to 25, 26 to 30, and 31 to 74. Education was classified into three levels (high school and below, bachelor’s degree, and postgraduate).

To calculate the body mass index (BMI), self-reported weight and height data were used, which correlate strongly with measured values.^(25)^ BMI was classified as: ≤18.49, underweight; 18.50–24.99, normal weight; 25.00–29.99, overweight; and ≥30.00 kg/m^2^, obesity. Physical activity (PA) was assessed using a questionnaire developed for a Swedish sample^(26)^ and previously translated into Spanish.^(27)^ Individuals were asked to identify the intensity with which they performed their main occupation and leisure-time activities. Leisure-time PA was categorised into ‘very light’, ‘light’, ‘moderate’, ‘active’, and ‘very active’. Occupational PA was categorised into ‘very light’, ‘light’, ‘moderate’, and ‘heavy’.

### Statistical analysis

Statistical analysis was performed using STATA software version 18. For descriptive analysis, absolute and relative frequencies of sociodemographic, BMI, and lifestyle factors were obtained. The prevalence of each DE behaviour and cognition was estimated. ANOVA or the Student’s *t*-test was used to compare the means of each DE indicator across sociodemographic characteristics, BMI, and lifestyle factors. Significance was considered when *p* ≤ 0.050.

Linear regression models were estimated in which the outcomes were the three DE indicators (negative body image, binge eating-compensation, and exercise as a negative experience). In all models, the following covariates were included: sex, age, education, BMI, leisure-time and occupational PA, and tobacco and alcohol use. These covariates were selected because being a woman^(28)^ or young,^(14)^ having a higher weight,^(29)^ leisure PA,^(9)^ and substance use^(14)^ are related to DE behaviours and thoughts. Considering that the frequency of DE differs between sexes,^(28)^ its interaction with the other covariates was included in the models. Only significant interactions were kept in the final model and plotted.

## Results

The most common DE behaviours and cognitions were feeling uncomfortable or insecure about body fat (74.7%), feeling ashamed of weight (57.6%), feeling uncomfortable or insecure about muscles (57.2%), feeling bad when their weight is measured (54.8%), and feeling like they have lost control over what they eat (51.0%) (Table 1). In contrast, the least common DE behaviours and cognitions were inducing vomiting (5.0%) or losing at least 7 kg in the last 3 months (5.4%). Ten out of fifteen indicators of DE were more frequent in women than in men.

Most patients were female (71.7%), aged 18–25 years (67.3%), with a bachelor’s degree (65.1%), normal weight or overweight (71.5%), moderate or very active in their leisure time (47.0%), or reported very light occupational PA (56.0%) (see Table 2). Two out of ten smoked, and six out of ten consumed alcoholic beverages.

**Table 2.** Sociodemographic characteristics and nutrition-related behaviours of patients from the nutritional care offices, Mexico city (*N* = 502)

Variables	Mean	SD
Age	26.7	0.5
Body mass index	26.6	0.2
	*n*	%
Sex		
Men	142	28.3
Women	360	71.7
Age, years		
18–20	144	28.7
21–25	194	38.6
26–30	63	12.6
31–74	101	20.1
Education		
Primary	6	1.2
Junior high school	7	1.4
High school and below	126	25.1
Bachelor	327	65.1
Postgraduate	36	7.2
Body mass index		
Underweight	23	4.6
Normal	182	36.3
Overweight	177	35.2
Obesity	120	23.9
Leisure-time physical activity		
Very light	94	18.7
Light	73	14.6
Moderate	104	20.7
Active	132	26.3
Very active	99	10.7
Occupational physical activity		
Very light	281	56.0
Light	94	18.7
Moderate	118	23.5
Heavy	9	1.8
Tobacco use	100	19.9
Alcohol use	307	61.2

*Note*: SD, standard deviation; *n*, absolute frequency in the sample.

Binge eating-compensation and negative body image scores were higher among women (compared to men), younger individuals, those with higher body weight, and those with very light or light leisure-time PA (see Table 3). Binge eating-compensation scores were higher among those with a bachelor’s degree than among those with a graduate degree. Exercise as a negative experience scores were higher among younger people, those with a bachelor’s degree, who had a normal weight or overweight, and those who engaged in higher intensity leisure-time PA. Scores on the three DE behaviours and cognitions did not differ by occupational PA, tobacco, or alcohol use.

**Table 3.** Disordered eating patterns according to patients’ sociodemographic characteristics and nutrition-related behaviours in patients from the nutritional care offices, Mexico city (*n* = 502)

		Binge eating-compensation		Negative body image		Exercise as negative experience	
Overall sample							
Mean ± standard deviation		1.75 ± 1.64		2.44 ± 1.38		1.26 ± 1.08	
Minimum/Maximum		0/6		0/4		0/3	
Covariates	*n*	Mean	*p*	Mean	*p*	Mean	*p*
Sex							
Men	142	1.17 ± 1.34	*0.000*	1.85 ± 1.41	*0.000*	1.20 ± 1.04	*0.460*
Women	360	1.97 ± 1.69		2.67 ± 1.30		1.28 ± 1.10	
Age, years							
18–20	144	2.20 ± 1.63	*0.000*	2.71 ± 1.23	*0.000*	1.62 ± 1.05	*0.000*
21–25	194	1.73 ± 1.67		2.51 ± 1.37		1.29 ± 1.08	
26–30	63	1.42 ± 1.43		2.43 ± 1.43		1.17 ± 1.09	
31 or more	101	1.32 ± 1.58		1.92 ± 1.47		0.74 ± 0.90	
Education							
High school and below	139	1.85 ± 1.65	*0.036*	2.47 ± 1.42	*0.462*	1.17 ± 1.66	*0.000*
Bachelor	327	1.77 ± 1.66		2.45 ± 1.37		1.36 ± 1.10	
Postgraduate	36	1.08 ± 1.25		2.16 ± 1.34		0.69 ± 1.00	
Body mass index							
Underweight	23	0.60 ± 0.94	*0.000*	2.26 ± 1.36	*0.000*	0.74 ± 0.92	*0.015*
Normal	182	1.55 ± 1.56		2.12 ± 1.39		1.36 ± 1.08	
Overweight	177	1.88 ± 1.65		2.48 ± 1.32		1.33 ± 1.11	
Obesity	120	2.08 ± 1.74		2.90 ± 1.03		1.10 ± 1.03	
Leisure-time physical activity							
Very light	94	2.29 ± 1.68	*0.005*	2.89 ± 1.28	*0.000*	0.88 ± 0.95	*0.000*
Light	73	1.78 ± 1.64		2.69 ± 1.39		0.86 ± 0.99	
Moderate	104	1.46 ± 1.58		2.38 ± 1.37		1.24 ± 1.11	
Active	132	1.64 ± 1.61		2.34 ± 1.34		1.45 ± 1.12	
Very active	99	1.65 ± 1.62		2.01 ± 1.41		1.67 ± 0.95	
Occupational physical activity							
Very light	281	1.76 ± 1.69	*0.974*	2.50 ± 1.36	*0.064*	1.21 ± 1.10	*0.299*
Light	94	1.72 ± 1.39		2.57 ± 1.39		1.25 ± 1.09	
Moderate and heavy	127	1.74 ± 1.57		2.19 ± 1.41		1.38 ± 1.03	
Tobacco use							
No	402	1.71 ± 1.62	*0.311*	2.40 ± 1.39	*0.129*	1.26 ± 1.08	*0.925*
Yes	100	1.90 ± 1.72		2.63 ± 1.35		1.27 ± 1.10	
Alcohol consumption							
No	195	1.70 ± 1.68	*0.598*	2.43 ± 1.35	*0.882*	1.25 ± 1.04	*0.873*
Yes	307	1.78 ± 1.62		2.44 ± 1.40		1.27 ± 1.11	

*Note:* Mean, crude means are reported.

After adjusting for other covariates, older individuals had lower scores on the three DE indicators (*B* = −1.11 for binge eating-compensation, *B* = −1.26 for negative body image, and *B* = −0.65 for exercise as negative experience) (Table 4). Individuals who engaged in leisure-time PA at higher intensity tended to have higher scores on the exercise as a negative experience scale (*B* = 0.42 for active and *B* = 0.63 for very active).

**Table 4.** Linear regression models with disordered eating patterns as outcomes and sociodemographic characteristics and nutrition-related behaviours as covariates in patients from the nutritional care offices, Mexico city (*N* = 502)

	Binge eating-compensation		Negative body image		Exercise as negative experience	
Covariates	B	*95CI*	B	*95CI*	B	*95CI*
Sex						
Women	0.44	−0.52, 1.40	0.62	−0.15, 1.39	0.19	−0.02, 0.39
Age, years						
21–25	−0.47	−0.81, −0.12	−0.26	−0.53, 0.01	−0.28	−0.51, −0.06
26–30	−0.89	−1.37, −0.42	−0.55	−0.93, −0.17	−0.32	−0.64, 0.00
31 or more	−1.11	−1.55, −0.68	−1.26	−1.61, −0.91	−0.65	−0.94, −0.36
Education						
Bachelor	−0.02	−0.34, 0.29	−0.02	−0.27, 0.24	0.18	−0.03, 0.38
Postgraduate	−0.44	−1.04, 0.17	−0.01	−0.50, 0.48	−0.18	−0.58, 0.22
BMI						
Underweight	−0.00	−1.16, 1.15	1.15	0.22, 2.07	−0.59	−1.03, −0.15
Overweight	0.78	0.17, 1.39	1.01	0.55, 1.50	0.07	−0.14, 0.29
Obesity	0.91	0.20, 1.62	0.75	0.18, 1.33	−0.00	−0.25, 0.24
BMI × sex						
Underweight × sex	−1.40	−2.81, −0.00	−1.50	−2.64, −0.36		
Overweight × sex	−0.35	−1.06, 0.36	−0.66	−1.23, −0.09		
Obesity × sex	−0.40	−1.22, 0.43	0.22	−0.45, 0.88		
Leisure-time physical activity						
Light	−1.07	−2.12, −0.02	−0.41	−1.26, 0.43	−0.03	−0.35, 0.28
Moderate	−1.10	−2.02, −0.17	−0.49	−1.23, 0.26	0.34	0.05, 0.64
Active	−1.19	−2.09, −0.29	−0.81	−1.53, −0.08	0.42	0.14, 0.71
Very active	−1.48	−2.36, −0.60	−1.43	−2.14, −0.72	0.63	0.33, 0.94
Leisure-time physical activity × sex						
Light × sex	0.72	−0.45, 1.89	0.37	−0.57, 1.13		
Moderate × sex	0.41	−0.63, 1.45	0.22	−0.62, 1.06		
Active × sex	0.55	−0.46, 1.57	0.44	−0.38, 1.26		
Very active × sex	1.09	0.06, 2.11	0.91	0.09, 1.74		

*Note:* The reference groups were men, individuals aged 18 to 20, those with a high school diploma or less, those of normal weight, and those with very light leisure-time physical activity. For each disordered eating variable, a linear regression model was estimated. In addition to the covariates listed in the first column, the three models were adjusted for occupational physical activity, and tobacco and alcohol use.

B, linear regression coefficients; 95 CI, 95% confidence intervals.

**Figure 1.**
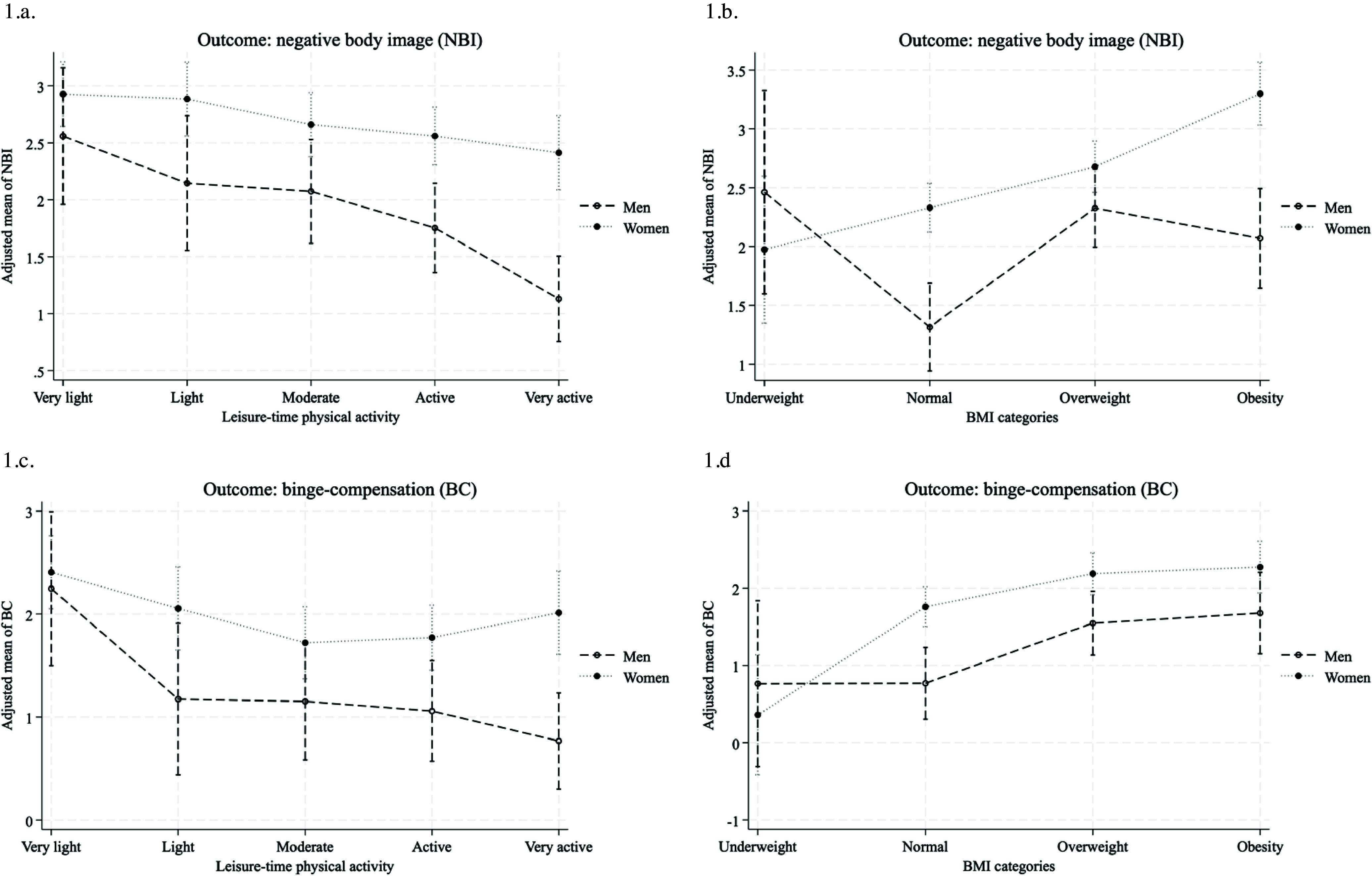
Interactions of sex with BMI or leisure-time physical activity to predict disordered eating in patients from the Nutritional Care Offices, Mexico City*. * Estimates derived from linear regression models adjusted by sex, age, education, body mass index, recreational physical activity, occupational physical activity, and tobacco and alcohol use.

In the models in which the outcome was negative body image, the interactions of sex with BMI (*B* = −1.50 and *B* = −0.66 for the interactions with underweight and overweight, respectively) and with leisure-time PA (*B* = 0.91 for the interaction with very active) were significant (see Table 4). In men, engaging in higher-intensity leisure-time PA was associated with lower scores in negative body image (Figure 1a). In women, this relationship was milder. In women, high BMI values were linearly related to higher scores of negative body image (Figure 1b). In men, the relation of BMI to negative body image was not linear: the lowest scores occurred among those with normal weight, whereas those with underweight, overweight, and obesity had higher scores.

In the models in which the outcome was binge eating-compensation, the interactions of sex with leisure-time PA (*B* = 1.09 for the interaction with very active) and BMI (*B* = −1.40 for the interaction with underweight) were significant (see Table 4). In men, engaging in leisure-time PA at higher intensity was associated with lower scores in binge eating-compensation (see Figure 1c). In women, this relationship was not observed; even those who engaged in more intense leisure-time PA had only slightly higher scores on binge eating-compensation than those who engaged in moderate or active PA. On the other hand, compared to men and women with normal weight, those with overweight or obesity had higher binge eating-compensation scores (Figure 1d). In these three weight groups (normal, overweight, and obesity) women had higher scores than men, while, in contrast, among people with underweight, women had lower scores than men.

## Discussion

The results of the current study show that most people who received care at the NCO project had thoughts, perceptions, or behaviours related to DE. The three forms of DE (negative body image, binge eating-compensation, and exercise as a negative experience) identified were more frequent in women, people with overweight or obesity, and young people. The practice of leisure-time PA was positively associated with exercise as a negative experience but negatively associated with negative body image.

The questionnaire applied includes various symptoms of DE, among which some are infrequent (vomiting and weight loss of 7 Kg or higher), while others (binge eating, loss of control in eating, and exercise as a compensatory measure) were reported by at least one-third of the patients. In our sample, the reported frequencies of exercising as a compensatory behaviour or feeling bad when not exercising were 43.0% and 44.0%, respectively. Similar indicators were less frequent in patients in primary care services: 16.9% and 13.0%, respectively.^(9)^ In US female veterans who attended primary care, the frequencies of objective or subjective binge eating and self-induced vomiting were 40.1% and 0.6%, respectively;^(10)^ while in our sample, these values were 35.7% and 5.0%, respectively. Furthermore, in NCO patients, between 5 and 7 out of 10 experienced some form of negative body image. By reporting and contrasting these figures (although the samples are not entirely comparable), we intend to show that, in our sample, a significant proportion of patients attending nutrition consultations exhibit DE behaviours and cognitions, consistent with previous studies. In addition, the frequency of DE in these patients may even be higher than that observed in the general population.

Measuring anthropometric dimensions is a routine activity in nutritional assessments, and prescribing restrictive eating plans is a frequent intervention.^(30)^ It has also been proposed that people should monitor their body weight to promote adherence and weight loss.^(31)^ However, our results and previous evidence^(32)^ suggest that some individuals who attend the nutrition clinic could experience these activities negatively. This situation makes it clear that in weight management, ‘health care practitioners are responsible for providing a psychologically safe, accessible, and respectful setting and empowering care’.^(30)^

In our sample, half of the patients reported symptoms related to binge eating (loss of control over eating). In addition, patients with overweight or obesity had higher levels of binge eating-compensation. In contrast, the most common interventions that nutritionists recommend for people with overweight or obesity are restrictive or hypocaloric diets.^(33)^ Considering our results, this may be inappropriate, since restrictive diets are a risk factor for the development of binge eating, which in turn promotes compensatory behaviours.^(34)^ In addition, restrictive diets are ineffective because they lead to significant weight loss in very few patients, and in most cases, there is weight regain.^(17,18)^ Unfortunately, in the traditional curriculum of the nutrition profession, little training is offered to identify and support patients who experience binge eating.

Exercise as a negative experience was also a common DE behaviour and cognition. Five out of ten patients feel uncomfortable or insecure about their muscles, and four out of ten reported using exercise to compensate for overeating or feeling negative emotions when missing a workout. In addition, exercise as a negative experience was associated with higher-intensity leisure-time PA. One way to interpret this association is that when people begin exercising more frequently, it becomes a source of worry; that is, there is a cause-and-effect relationship. Another way to interpret this association is that increased exercise and worry about it are two dimensions of the same phenomenon. A longitudinal study could help clarify these hypotheses. On the other hand, these findings suggest that cultural pressures centred on physical appearance have expanded. In previous decades, pressure focused on achieving a slim physique through restrictive diets (‘the diet culture’). Now, a new pressure has been added: to gain muscularity or muscle tone through exercise. For this reason, the concept of the ‘culture of the perfect body’ more accurately captures the experiences of patients seeking a nutrition consultation.

Our results confirm that women and individuals with higher body weight more frequently experience DE.^(28,29)^ This is significant because most people who attend weight-related nutrition consultations have both characteristics. Women with a normal weight in our sample presented higher levels of negative body image compared to men in the same weight group. Furthermore, in men, leisure-time PA conferred protection for negative body image and binge eating-compensation behaviours, which did not occur in women. Taken together, these results are consistent with the ‘objectification’ theory,^(28)^ according to which women are considered sexual objects by men. Therefore, women learn to try to modify their bodies to meet the demands of the ‘perfect body’. The result is that women are more concerned about their body image, even when they are at a normal weight. During consultations, nutritionists could promote a discussion that makes evident that people’s desire to modify their bodies is a product of socio-cultural pressures.

**Limitations**. One of the main limitations of the present study is that the participants are patients who received care through a university project. This is evident in the predominance of young people. Despite this, the authors hypothesise that similar results to those reported herein would be observed in other care centres where weight management is a common reason for consultation. This is expected because the cultural norms that foster the development of DE are widespread in Western societies.^(35)^ Future studies should confirm or reject this hypothesis.

Although the tool used to screen DE was based on items from previously validated inventories, new questions derived from clinical practice were also included. In addition, the change from Likert-type to dichotomous answer options could result in a loss of information. The questions have not been validated to identify DE or ED. Another limitation of the tool is that it did not include specific questions about dietary restriction. Possibly, the item on weight loss was left as an independent factor because it identifies restrictive behaviours. More research is required to validate the developed screening questionnaire and replicate the results in other settings. Finally, the cross-sectional design of the study precludes us from drawing causal conclusions or making assertions about the direction of the associations.

## Conclusion

We found that DE is a frequent problem among patients motivated to change their weight or body composition. Our results make it evident that thoughts and behaviours related to restrictive practices for weight loss are not the only common DE, but concerns about muscularity and negative experiences with exercise are also frequent. There are higher-risk groups (such as women, younger or higher-weight individuals, and those who are more active during leisure time) that deserve special attention.

Our findings suggest that nutrition professionals should systematically screen for DE in adult weight-management care settings. Nutritionists should develop the required skills (reflective listening,^(30)^ unconditional acceptance,^(36)^ and compassionate detachment) to discuss DE with their patients, as this topic can be sensitive for both parties. Based on the screening results, nutritionists should define appropriate nutritional assessments and interventions. For example, professionals should weigh the benefits and risks^(37)^ of taking body measurements from people with DE. For patients with DE, interventions such as intuitive eating or health at every size may be better options.^(30)^
